# Prevalence and risk factors of hypertension among college freshmen in China

**DOI:** 10.1038/s41598-021-02578-4

**Published:** 2021-11-29

**Authors:** Qingqing Jiang, Qiumei Zhang, Tiantian Wang, Qiqi You, Chun Liu, Shiyi Cao

**Affiliations:** 1 School of Public Health, Tongji Medical College, Huazhong University of Science and Technology, No. 13 Hangkong Road, Wuhan, 430030 Hubei China; 2 Hospital of Jianghan University, Wuhan, 430056 Hubei China; 3 Division of Cardiothoracic and Vascular Surgery Department, Tongji Hospital, Tongji Medical College, Huazhong University of Science and Technology, No. 1095 Jiefang Avenue, Wuhan, 430030 Hubei China

**Keywords:** Cardiology, Health care, Risk factors

## Abstract

Hypertension is the leading single contributor to all-cause death and disability worldwide. However, there is scarce evidence on the prevalence and risk factors of hypertension for Chinese youth. This study aimed to investigate the prevalence of hypertension among Chinese college freshmen and to identify the influencing factors. We conducted a cross-sectional study of all freshmen from 2015 to 2017 at a university in Wuhan, China by physical examination and standard-structured questionnaire. The Pearson chi-square test was used to compare categorical variables. Forward stepwise logistic regression method was used in the multivariate analysis to identify independent predictors of hypertension in youth. A total of 12,849 participants were included, and the prevalence of hypertension of Chinese college freshmen was 4.3% (7.9% in men and 1.6% in women). Men had a higher risk of hypertension than women (odds ratio [OR]: 5.358, 95% confidence interval [CI]: 4.345–6.607, *P* < 0.001). Obese people were more likely to develop hypertension than those with normal body mass index (OR: 10.465, 95% CI: 8.448–12.964, *P* < 0.001). People with elevated resting heart rate (RHR) had a higher prevalence of hypertension (OR: 4.987, 95% CI: 3.641–6.832, *P* < 0.001). Staying up late (OR: 2.957, 95% CI: 2.482–3.523, *P* < 0.001), physical inactivity (OR: 4.973, 95% CI: 4.141–5.972, *P* < 0.001), living in urban district (OR: 1.864, 95% CI: 1.493–2.329, *P* < 0.001) and family history of cardiovascular diseases (CVDs) (OR: 2.685, 95% CI: 2.108–3.421, *P* < 0.001) were related to higher prevalence of hypertension in youth. Male, obesity, elevated RHR, physical inactivity and family history of CVDs were identified as important risk factors of hypertension in youth. These risk factors should be given more attention when designing and implementing the interventional programs.

## Introduction

Cardiovascular diseases (CVDs, including coronary heart disease, heart failure, stroke, myocardial infarction and atrial fibrillation etc.) are the most common non-communicable diseases globally and responsible for estimated 17.9 million deaths each year^1,2^, which caused an increasing burden to the society and families. Although CVDs are usually detected in the elderly, the disease development process that cannot be observed obviously may occur at a younger age, and there is an increasing tendency of mortality among the young^3,4^. Hypertension, as the most common preventable risk factor for CVDs, is the leading single contributor to all-cause death and disability worldwide^5^. Increasing studies have provided ample evidence that some adult hypertension develops in childhood, meaning that children and adolescents with elevated blood pressure tend to end up as adults with recognizable hypertension^6,7^. From a public health point of view, the prevention and control of hypertension are essential to maintain and promote human health, particularly in childhood.

In China, the prevalence of hypertension among youth rose from 1.6%^8^ in 2004 to 4.0%^9^ in 2015, but there is scarce evidence on the prevalence and risk factors of hypertension for Chinese youth. The aim of the cross-sectional study was to investigate the prevalence of hypertension of college freshmen as well as its influencing factors, which would provide scientific bases for the primary prevention of CVDs. We hope to awaken the students’ attention to take active measures to keep their blood pressure within the normal range.

## Methods

### Ethics statement

Our study was approved by the Research Ethics Committee in Tongji Medical College, Huazhong University of Science and Technology, Wuhan, China. All study subjects understood the purpose of this survey and signed informed consent forms. All identifiable personal information was removed for privacy protection. All methods were performed in accordance with the Declaration of Helsinki.

### Settings and study subjects

The cross-sectional study was an investigation of the prevalence of hypertension among college freshmen and the influencing factors associated with it. We investigated all the freshmen of a university in Wuhan, China from 2015 to 2017, who had graduated from high schools all over China. Finally, we identified 12,849 college freshmen to take part in our survey, not including students who were demoted to the freshman from other grades.

### Data collection

Organized by the college as a unit, college freshmen had orderly physical examinations and standard-structured questionnaires. Physical examination included height, weight, resting heart rate (RHR), systolic blood pressure (SBP) and diastolic blood pressure (DBP) and so on. Based on literature research and expert consultation, standard-structured questionnaires were designed and distributed to the participants. The contents of the questionnaire were as follows: enrollment year, gender, age, residence, region, whether they exercised at least 150 min per week, whether they slept less than six hours per day, and whether they had a family history of CVDs (first-degree relatives had a history of CVDs). Height and weight were measured using a calibrated scale and a stadiometer, with the participants barefoot and in light clothing. After the participants had been seated and rested for five minutes, blood pressure and RHR were measured three times by an Omron HEM-705P arm-type electronic sphygmomanometer, with an interval of no less than two minutes between every two measurements. The average of the last two readings was taken in the analysis.

### Definition of hypertension

According to the diagnostic criteria of *Chinese Guidelines for The Prevention and Treatment of Hypertensio*n^10^, we divided SBP < 120 mmHg and DBP < 80 mmHg as normal blood pressure, 120 mmHg ≤ SBP < 140 mmHg and/or 80 mmHg ≤ DBP < 90 mmHg as normal high blood pressure, and SBP ≥ 140 mmHg and/or DBP ≥ 90 mmHg as hypertension. Our current study aimed to explore the factors affecting hypertension, so we combined normal blood pressure and normal high blood pressure as the contrary of hypertension.

### Explanatory variables

Most of the variables were self-explanatory, but a few needed explanations. Based on the formula for calculating the body mass index (BMI), we calculated the value of BMI of each participant and classified them according to the diagnostic criteria for overweight and obesity in Chinese adults^11^. We classified BMI between 18.5 and 23.9 kg/m^2^ as normal weight, BMI between 24 and 27.9 kg/m^2^ as overweight, and BMI ≥ 28 kg/m^2^ as obesity; the classification standards for minors referred to Table 1. According to the normal range of RHR , RHR < 60 beats/min was defined as bradycardia, RHR between 60 beats/min and 100 beats/min was defined as normal heart rate, and RHR > 100 beats/min was defined as tachycardia^12^. We defined RHR greater than 100 beats/min as elevated RHR, and the others were the contrary. Adequate physical activity was defined as more than 150 min of exercise per week. Stay up late was defined as less than 6 h of sleep duration per day.

**Table 1:** Guidelines for the prevention and control of overweight and obesity in school-age children in China.

Age, years old	Overweight (BMI, kg/m^2^)		Obesity (BMI, kg/m^2^)	
	Male	Female	Male	Female
15	23.1	23.4	26.9	26.9
16	23.5	23.7	27.4	27.4
17	23.8	23.8	27.8	27.7

### Statistical analysis

Data from physical examinations and questionnaires were recorded into Excel 2016 and statistical analysis was performed by the SPSS 22. The quantitative data were presented in form of mean ± standard deviation (SD), and the qualitative data were presented in form of percentage. The one-way ANOVA and the t test were performed to evaluate continuous data. Pearson chi-square tests were used to compare categorical variables. Forward stepwise logistic regression method was used in the multivariate analysis to identify independent predictors of hypertension among college freshmen using odds ratio (OR) with 95% confidence interval (95% CI). Variables were retained in the model based on the likelihood ratio χ^2^ statistic at *P*-values < 0.05. All comparisons were two-tailed, and *P*-values less than 0.05 were considered statistically significant.

## Results

### Demographic characteristics of the participants

A total of 12,849 participants were enrolled in the analysis, including 7197 females and 5652 males (Table 2). The mean ± SD of SBP and DBP were 119.4 ± 12.5 mmHg and 70.9 ± 8.9 mmHg, respectively. The participants from the Western, Midland and Eastern regions accounted for 7.5%, 79.8% and 12.7%, respectively, and there was significant discrepancy of mean blood pressure in various region (*P* < 0.05). 72.3% of the freshmen lived in urban district before entering the college. 12.8% of the college freshmen were overweight, and 5.5% of them were obesity with an average SBP of 131.3 mmHg. 67.6% of the respondents usually exercised ≥ 150 min per week, and the students with physical inactivity had higher SBP and DBP (*P* < 0.05). 19.5% of the students habitually stayed up late, and their average SBP was 125.2 ± 11.7 mmHg, which was significantly higher than that of the students with regular sleep (*P* < 0.05). Students with a family history of CVDs were found to account for 6.7% of the college freshmen, and their average DBP was 72.0 ± 10.0 mmHg. The participants who had elevated RHR had higher SBP (127.3 ± 15.0 mmHg) and DBP (78.8 ± 10.2 mmHg) than the students whose RHR were under 100 beats/min (*P* < 0.05).

**Table 2:** Socio-demographic characteristics and blood pressure of the participants.

Variables	*N*	%	Blood pressure (mmHg)			
			Systolic blood pressure mean ± standard deviation	*P*-value	Diastolic blood pressure mean ± standard deviation	*P*-value
Total	12,849	100.0	119.4 ± 12.5		70.9 ± 8.9	
**Year**				< 0.001		< 0.001
2015	4436	34.5	118.8 ± 12.3		70.4 ± 9.1	
2016	4128	32.1	118.5 ± 12.4		71.1 ± 8.3	
2017	4285	33.4	121.1 ± 12.6		71.3 ± 9.1	
**Gender**				< 0.001		< 0.001
Male	5652	44.0	125.7 ± 11.1		72.6 ± 9.1	
Female	7197	56.0	114.5 ± 11.2		69.6 ± 8.4	
**Residence**				< 0.001		< 0.001
Urban	9289	72.3	120.0 ± 12.6		71.4 ± 8.9	
Rural	3560	27.7	118.0 ± 12.0		69.6 ± 8.8	
**Region**				< 0.001		< 0.001
West	959	7.5	116.0 ± 12.1		68.3 ± 8.3	
Midland	10,259	79.8	119.8 ± 12.5		71.2 ± 9.0	
East	1631	12.7	119.3 ± 12.3		70.3 ± 8.4	
**Body mass index**				< 0.001		< 0.001
Normal	10,497	81.7	117.6 ± 11.9		69.9 ± 8.4	
Overweight	1646	12.8	126.1 ± 11.4		74.0 ± 9.2	
Obesity	706	5.5	131.3 ± 11.1		78.6 ± 9.8	
**Adequate physical activity**				< 0.001		< 0.001
Yes	8687	67.6	115.5 ± 11.3		69.0 ± 7.9	
No	4162	32.4	127.7 ± 10.5		74.8 ± 9.5	
**Stay up late**				< 0.001		< 0.001
Yes	2511	19.5	125.2 ± 11.7		73.4 ± 9.3	
No	10,338	80.5	118.0 ± 12.2		70.3 ± 8.7	
**Family history of cardiovascular diseases**				0.565		0.001
Yes	856	6.7	119.2 ± 13.9		72.0 ± 10.0	
No	11,993	93.3	119.5 ± 12.3		70.8 ± 8.8	
Resting heart rate				< 0.001		< 0.001
≤ 100	12,555	97.7	119.3 ± 12.3		70.7 ± 8.8	
> 100	294	2.3	127.3 ± 15.0		78.8 ± 10.2	

Table 3 shows the prevalence of hypertension and the comparison between groups according to different characteristics of college freshmen. Overall, 558 participants (4.3%) had hypertension. Pearson chi-square tests indicated that hypertension was associated with students’ gender, residence, and BMI (*P* < 0.001). Notably, students with elevated RHR, with physical inactivity, students who slept late and who had a family history of CVDs were more likely to have hypertension (*P* < 0.001).

**Table 3:** The association between hypertension and socio-demographic characteristics of college freshmen.

Variables	Hypertension (*N*, %)		χ^2^	*P*-value
	No	Yes		
Total	12,291 (95.7)	558 (4.3)	…	…
**Year**			109.4	< 0.001
2015	4303 (97.0)	133 (3.0)		
2016	4003 (97.0)	125 (3.0)		
2017	3985 (93.0)	300 (7.0)		
**Gender**			302.8	< 0.001
Male	5207 (92.1)	445 (7.9)		
Female	7084 (98.4)	113 (1.6)		
**Residence**			31.0	< 0.001
Urban	8828 (95.5)	461 (5.0)		
Rural	3463 (97.3)	97 (2.7)		
**Region**			10.7	0.005
West	937 (97.7)	22 (2.3)		
Midland	9793 (95.5)	466 (4.5)		
East	1561 (95.7)	70 (4.3)		
**Body mass index**			650.9	< 0.001
Normal	10,220 (97.4)	227 (2.6)		
Overweight	1521 (92.4)	125 (7.6)		
Obesity	550 (77.9)	156 (22.1)		
**Adequate physical activity**			353.4	< 0.001
Yes	8513 (98.0)	174 (2.0)		
No	3778 (90.8)	384 (9.2)		
**Stay up late**			160.2	< 0.001
Yes	2286 (91.0)	225 (9.0)		
No	10,005 (96.8)	333 (3.2)		
**Family history of cardiovascular diseases**			68.9	< 0.001
Yes	771 (90.1)	85 (9.9)		
No	11,520 (96.1)	473 (3.9)		
Resting heart rate			122.5	< 0.001
≤ 100	12,048 (96.0)	507 (4.0)		
> 100	243 (82.7)	51 (17.3)		

### Factors associated with hypertension of college freshmen

**Figure 1:**
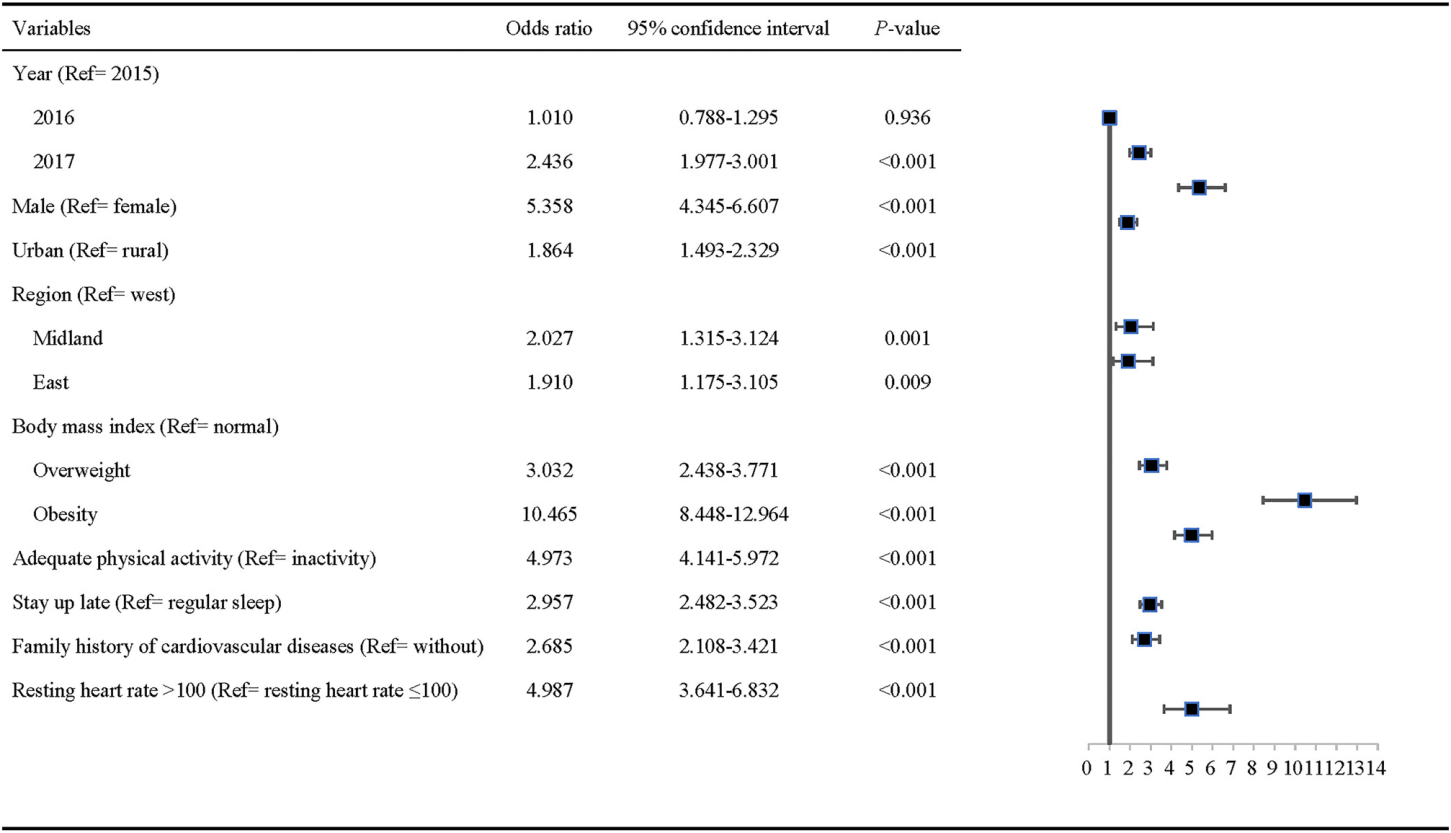
Factors associated with hypertension of college freshmen in multivariable logistic regression model.

Figure 1 presents the factors associated with participants’ hypertension by multivariable logistic regression model. The prevalence of hypertension was higher in men than in women (OR: 5.358, 95% CI: 4.345–6.607, *P* < 0.001). Freshmen who lacked of regular exercise were more likely to have hypertension (OR: 4.973, 95% CI: 4.141–5.972, *P* < 0.001). The college freshmen who came from urban district had a higher risk (OR: 1.864, 95% CI: 1.493–2.329, *P* < 0.001) of hypertension compared with students from rural district. People with a family history of CVDs were more likely to have hypertension (OR: 2.685, 95% CI: 2.108–3.421, *P* < 0.001). People who stayed up late had a higher risk of hypertension (OR: 2.957, 95% CI: 2.482–3.523, *P* < 0.001). Obese students were more likely to develop hypertension than students with normal BMI (OR: 10.465, 95% CI: 8.448–12.964, *P* < 0.001). When the RHR was more than 100 beats per minute, the risk of hypertension would increase in youth (OR: 4.987, 95% CI: 3.641–6.832, *P* < 0.001). Students from the middle of the country had a higher risk of hypertension (OR: 2.027, 95% CI: 1.315–3.124, *P* < 0.001) compared to western students.

## Discussion

Hypertension is a global epidemic and has become an increasingly important medical problem in children and adolescents. More and more researches on hypertension in adolescents remind us to strengthen the prevention and control of hypertension in the special population. However, information on the risk factors of hypertension in youth around the age of 18 years was limited. This study included 12,849 college freshmen with a mean age of 18.5 years, and we found that the prevalence of hypertension among freshmen in the university was similar to that of adolescent boys in Delhi, India^13^. Perhaps the reason was that both countries are developing countries, and a previous study had showed that the prevalence of hypertension in low- and middle-income countries has surpassed that in high-income countries^14^. Hypertension is an important risk factor for a variety of health conditions, such as CVDs, stroke, and kidney failure. Nevertheless, this burden is unevenly distributed in society, with higher rates of hypertension in men than in women^15^, which was in line with our findings. The reason may be that men were more likely to lack healthy eating and exercise habits. Women usually are more aware of and are more active in managing and treating their blood pressure, which was consistent with a previous research on women’s health-seeking behaviors^16^. The prevalence of hypertension was significantly lower in rural areas than that in urban areas, which was consistent with some relevant studies^17,18^. It was perhaps because that students from different residences had different eating habits and study pressure. People are prone to hypertension in stressful situations. Students from the middle land of the country had the highest rate of hypertension, which may be due to a prevalent excessive salty diet.

In recent decades, many epidemiological studies have reported a positive correlation between overweight and high blood pressure in pediatric populations^6,19^, and reported that obesity was a major risk factor for essential hypertension^20^. In our study, compared with students of normal BMI, those who were overweight or obese had a significantly higher prevalence of hypertension. The obese people were 10.5 times more likely to have hypertension than people of normal BMI, which was consistent with previous reports^21,22^. An epidemiological study showed that abdominal fat accumulation was associated with a higher risk of CVD incidence and mortality ^23^. On the one hand, this is possibly related to their sedentary lifestyle, increased fat content in their diet and decreased physical activities. On the other hand, the development of hypertension caused by obesity can occur via multiple mechanisms, such as insulin resistance, adipokine alterations, inappropriate sympathetic nerve function and renin–angiotensin–aldosterone system activation, structural and functional abnormalities in the kidney, heart and vascular changes, and immune maladaptation^20,24^. Uric acid and incretin or dipeptidyl peptidase 4 activity alteration also contribute to the development of hypertension in the context of obesity^24^. In addition, using BMI alone to diagnosis obesity would lead to about half of patients with abdominal obesity being missed.

Doing exercise is considered one of the most effective methods to control the weight of college students. Physical activity can not only reduce the subcutaneous and visceral fat of obese people, but also play a positive role in promoting cardiac function, autonomic nervous system and immune ability. This study showed that the detection rate of hypertension among college freshmen who do not participate in exercise regularly was significantly higher than that among those who exercised regularly. Physical exercise is a protective factor of hypertension. Owen et al. found that 10% increase in physical activity among adolescents can significantly reduce the risk of obesity and CVDs^25^. Moderate exercise was good for health and heart function. However, it did not mean that excessive exercise was better. Over the last several years, sudden deaths of trained athletes were usually associated with immoderate exercise, which have become highly visible events fueled by news media reports^26^. Therefore, moderate exercise is the best option.

Correlation analysis shows that blood pressure was most closely related to RHR, which was consistent with previous research results^27^. The detection rate of hypertension was 17.3% in students with elevated RHR, and the elevated RHR was also a risk factor for hypertension. The value of RHR reflected the balance between autonomic nerves and the function of heart. The acceleration of RHR could increase the fluctuation of blood flow, change the velocity and direction of blood flow, and cause damage to the vascular endothelium. The lipid then seeped into the lining of the blood vessels and promoted the development and progression of atherosclerosis^28^, which was the pathological basis of CVDs. An increase of 10 beats per minute in heart rate was associated with at least 20% increase in the risk of cardiac death^29,30^. Therefore, RHR control is an important target for the prevention of hypertension, which could reduce the risk of target organ damage and adverse cardiovascular events in patients with hypertension.

We found that 9.0% of the freshmen who stayed up late had been diagnosed with hypertension, which was much higher than the proportion of hypertension among the freshmen who had regular sleep (3.2%). Previous studies had shown that sleep deprivation was associated with high blood pressure^31,32^. Staying up late was a very common phenomenon among college students, because they were more relaxed academically and could spend more time to surf the internet after the college entrance examination. In recent years, the use of smart phones has become more and more popular in Chinese adolescents. The overall prevalence of Internet addiction was 26.50% among adolescents in China^33^, which is much higher than that in other Asian countries (range: 6.2–21.2%)^34,35^. Internet addiction was found to affect sleep quality and lead to negative impact on health-related quality of life in adolescents^35^. Students with poor quality sleep may be at a higher risk of elevated blood pressure that could lead to CVDs in adolescents^32^. The possible explanation was that insufficient sleep put the students in a state of mild stress, and the psychological stress could act as a stressor on the human body and produce a stress response. Moreover, insufficient sleep acts on target organs through the thalamus-pituitary-adrenal system, which makes the sympathetic nervous system overactive, increases the release of catecholamines, and causes an increase in blood pressure.

The majority (90–95%) of the hypertensive patients have a highly heterogeneous primary hypertension with a multifactorial gene-environment etiology^36^. Our results showed that the family history was an important risk factor for hypertension. People with hypertension often have a positive family history, with the heritability (a measure of how much of the variation in a trait is due to variation in genetic factors) estimated between 35 and 50% in the majority of studies^37,38^.

### Strengths and limitations

The strengths of the current study were as follows. Firstly, the included participants were about 18 years, who were easily exposed to disease-related risk factors but did not have adequate disease prevention awareness. This investigation highlighted the early prevention of hypertension and played an early warning role in the occurrence of CVDs. Secondly, the sample size of this study was relatively large, and the results were relatively reliable. Thirdly, the sample is nationally representative to some extent, for all participants were just graduated from high schools all over the country.

Several limitations of this study should be listed. First of all, we failed to track the changes in hypertension during the following years of college, and the cross-sectional nature of the study limits drawing of inferences about causation. The second limitation was that we did not assess some other potential factors associated with hypertension, such as daily eating habits (especially salt intake), usage of stimulants and depression. Thirdly, not being able to collect data about use of antihypertensives means that the current study might have underestimate true prevalence of hypertension.

## Conclusions

In summary, the detection rate of hypertension in male was significantly higher than that in female of college freshmen in China. Obesity, physical inactivity, staying up late and elevated RHR were identified as important modifiable risk factors of hypertension in youth. It is necessary to change the lifestyle of these vulnerable groups and implement intervention plans in the early stage, especially for obese and inactive men.

## Data availability 

Data are available purposes upon request.
